# Case Report: DSM–5 misses an edge case in tic disorders nosology

**DOI:** 10.12688/f1000research.23991.1

**Published:** 2020-06-03

**Authors:** Kevin J. Black

**Affiliations:** 1 Departments of Psychiatry, Neurology, Radiology and Neuroscience, Washington University in St. Louis School of Medicine, St. Louis, MO, 63110-1093, USA

**Keywords:** Tourette syndrome, tic disorders, nosology, DSM-5, case report

## Abstract

A boy with multiple phonic tics, one lifetime motor tic, and no impairment or marked distress does not meet criteria for any DSM–5 tic disorder diagnosis. The next version of the Diagnostic and Statistical Manual should adjust the criteria for Tourette's Disorder and/or for "other specified tic disorder" and "unspecified tic disorder."

## Introduction

Over time, tic disorder nosology has changed ^1^. The current research criteria changed modestly with the adoption of DSM–5 ^2^. I describe a case of a child with motor and vocal tics that demonstrates a gap in DSM–5’s diagnostic criteria for tic disorders ^3^.

## Case report

At age 9, a right-handed, non-Hispanic white boy and his mother participated in a thorough research assessment as part of the New Tics study ^4^. The study was approved by the Washington University in St. Louis Human Research Protection Office, IRB ID #201109157, and his mother gave informed consent. This visit included questionnaires, K-SADS parent and child interviews, history of illness, neurological exam, 30 minutes of observation of the child alone via video, and YGTSS rating. His mother was a reliable informant, an elementary school teacher well informed about tics, and his father was a physician. His mother dated his tic onset to 8 months ago, at age 8. The child had seasonal allergies but the phonic tics were present when he had no allergy symptoms, and the tics did not respond to cetirizine. He was taking extended-release mixed amphetamine salts (40 mg daily) for ADHD with good response. K-SADS diagnoses were specific phobia, past social anxiety disorder, past nocturnal enuresis, predominantly inattentive ADHD since age 6, and provisional tic disorder.

Neurological exam was normal except for a medium-loud snort occurring once during the exam. He had simple phonic tics (sniff or snort, cough, clear throat), one motor tic (biting lower lip softly, seen during video observation) and no complex tics. He bit his nails sometimes since early childhood, but this was not counted as a tic given the timing and its high prevalence among young children ^5^. He also had one probable simple motor stereotypy (rarely shook his hands up and down near his chest before sports or social events since age 5 or younger; he said “I like doing that” and said it didn’t feel like his tics; seen only once in over an hour of observation). YGTSS scores were: motor tics 5, phonic tics 12, impairment 10.

He returned at 12 months after tic onset. The stimulant continued at the same dose, now without an antihistamine. The same tics continued within the past week, though not every day, but no tics were observed at the visit. YGTSS scores were: motor tics 4, phonic tics 6, impairment 0. By 24 months after tic onset, he was taking no medications. The lip biting had disappeared but the phonic tics continued. He reported that “they’re kind of annoying and I would like them to go away,” but he did not have marked distress and the tics did not affect self-esteem, family life, friendships or school functioning. No tics were observed during thorough history and a neurological examination, but sniffing, coughing and forceful nasal exhalations were observed by video when he was alone in the room. No motor tics were observed. YGTSS scores were: motor tics 0, phonic tics 9, impairment 0. Diagnostic Confidence Index score was 35 ^6^.

## Discussion

This boy has a fairly typical history for mild Tourette syndrome, except that he has only one motor tic. (Other clinicians may choose to count the nail biting or hand shaking as tics, but for the present discussion the main point is that some children will have a presentation with vocal tics and one motor tic.) The DSM–5 criteria for Tourette’s Disorder require “multiple” motor tics ^2^. The criteria for Persistent (Chronic) Vocal Tic Disorder exclude patients who have experienced both motor and vocal tics. At the follow-up visits, the duration of ticcing excludes Provisional Tic Disorder, and the history and examination provided no evidence for causation by a substance or non-psychiatric illness. The residual categories, Other Specified Tic Disorder and Unspecified Tic Disorder, require “clinically significant distress or impairment in social, occupational, or other important areas of functioning.” This last criterion is shared with most DSM–5 disorders, but since DSM–IV–TR it has been omitted for Tourette’s Disorder ^7^. Thus this boy does not meet DSM–5 criteria for any tic disorder. Woods and Thomsen addressed the situation in which a patient has vocal tics and exactly one motor tic, and concluded that “the requirement that multiple motor tics exist seems arbitrary and unnecessarily exclusive” ^8^. A DSM–5 work group discussed the nosological issues in detail ^7^. They retained the “multiple” motor tic requirement for Tourette’s Disorder, and did not propose reinstating the impairment or distress criterion in the “not otherwise specified” diagnosis. Roessner and colleagues provided critical feedback on the proposed criteria for tic disorders, but also assumed the impairment or distress criterion would be absent for all tic disorders ^9^. This change appears to have been inadvertent.

## Conclusions

There is no clinical import for this child, as his symptoms bother him only slightly. But this case demonstrates that the current DSM–5 criteria inadvertently provide no diagnosis in this case, which may occasionally affect research on tic disorders. The exclusion of one motor tic from both Tourette’s Disorder and Persistent Vocal Tic Disorder leaves a gap. The residual diagnostic categories no longer cover this gap since DSM–5 requires the “impairment or distress” criterion for them, though that requirement may have been accidental. I propose that future revisions omit it for all tic disorders. I also agree with Woods and Thomsen’s opinion that one motor tic and multiple phonic tics is best described as Tourette syndrome.

## Data availability

All data underlying the results are available as part of the article and no additional source data are required.

## Consent

Written informed consent for publication of their clinical details was obtained from the parent of the patient.
