# Supplementary material for: Case Report: DSM–5 misses an edge case in tic disorders nosology
Source: F1000Res. 2020 Jun 3;9:505. [Version 1] doi: 10.12688/f1000research.23991.1 (PMC7309051; doi:10.12688/f1000research.23991.1)
Supplement: Peer review history

# Peer review: Reviewer response for version 1

**DOI:** 10.5256/f1000research.26466.r64376

**Published:** 2020-06-17

**Authors:** Carol Mathews

**Affiliations:** 1 Department of Psychiatry, Genetics Institute, University of Florida, Gainesville, FL, USA

This case report discusses a 9 year old boy who presents with persistent, multiple vocal tics, and one motor tic.  Based on history and physical exam, he clearly meets criteria for multiple chronic tics, but has no demonstrated impairment. I would argue that he has mild distress, as evidenced by the fact that he finds them annoying, but from a practical perspective, they do not functionally impact his life. The author argues that this child's tics would not fit into any of the current DSM-5 tic disorder classifications, and most specifically, that he would not fit into either of the two that are closest to his presentation--Tourette's Disorder, because he does not have multiple motor tics, or persistent vocal tic disorder, because he does have one motor tic. This highlights a gap in the current diagnostic criteria (even leaving out the question of whether impairment should be necessary for a diagnosis, which is also addressed). Genetic and emerging clinical data suggest that chronic tics, whether primarily motor,vocal, or a mix of both, emerge from the same underlying genetic causes, and thus are likely to be manifestations of the same disorder. The argument is made, appropriately, for a revisiting of the tic disorder diagnoses in the DSM, to account for this gap, and perhaps also to incorporate the current genetic information when determining the disorder definitions for the next update.

Are enough details provided of any physical examination and diagnostic tests, treatment given and outcomes?

Yes

Is the case presented with sufficient detail to be useful for other practitioners?

Yes

Is sufficient discussion included of the importance of the findings and their relevance to future understanding of disease processes, diagnosis or treatment?

Yes

Is the background of the case’s history and progression described in sufficient detail?

Yes

Reviewer Expertise:

Tourette syndrome genetics, OCD genetics

I confirm that I have read this submission and believe that I have an appropriate level of expertise to confirm that it is of an acceptable scientific standard.

## References

[1] : Interrogating the Genetic Determinants of Tourette’s Syndrome and Other Tic Disorders Through Genome-Wide Association Studies. *American Journal of Psychiatry*.2019;176(3) : 10.1176/appi.ajp.2018.18070857 217-227 10.1176/appi.ajp.2018.18070857 PMC667725030818990

[2] : Polygenic Risk Scores Derived From a Tourette Syndrome Genome-wide Association Study Predict Presence of Tics in the Avon Longitudinal Study of Parents and Children Cohort. *Biological Psychiatry*.2019;85(4) : 10.1016/j.biopsych.2018.09.011 298-304 10.1016/j.biopsych.2018.09.011 30424865PMC6342633

# Peer review: Reviewer response for version 1

**DOI:** 10.5256/f1000research.26466.r64373

**Published:** 2020-06-11

**Authors:** Andrea Cavanna

**Affiliations:** 1 Department of Neuropsychiatry, BSMHFT, University of Birmingham, Birmingham, UK

Kevin Black’s case report highlights a possible hole in the current classification scheme for tic disorders.

 The author describes the clinical presentation of a 9-year-old boy who has a fairly typical history for mild Tourette syndrome, except that he has only one motor tic. Since he does not have an impairment or marked distress, he does not meet current diagnostic criteria for any DSM-5 tic disorder diagnosis.

 This interesting report is useful, as it hints at two possible solutions, not mutually exclusive. Firstly, it would be appropriate to consider relaxing the current criteria for the categories “Other Specified Tic Disorder” and “Unspecified Tic Disorder”, by reviewing the persisting criterion of “clinically significant distress or impairment in social, occupational, or other important areas of functioning”. Secondly, on a more general level, this case report calls into question the rationale for the distinction between motor and vocal/phonic tics. It is hoped that future classification systems will be increasingly more informed by advances in research that would allow them to “carve nature at its joints”.

Are enough details provided of any physical examination and diagnostic tests, treatment given and outcomes?

Yes

Is the case presented with sufficient detail to be useful for other practitioners?

Yes

Is sufficient discussion included of the importance of the findings and their relevance to future understanding of disease processes, diagnosis or treatment?

Yes

Is the background of the case’s history and progression described in sufficient detail?

Yes

Reviewer Expertise:

Behavioural neurology and neuropsychiatry

I confirm that I have read this submission and believe that I have an appropriate level of expertise to confirm that it is of an acceptable scientific standard.

# Response

**Published:** 2020-06-11

**Authors:** Kevin J Black

**Affiliations:** Department of Psychiatry, Washington University in St Louis, USA

I thank Dr. Cavanna for the review. I agree with his comment about the artificial and historical nature of the distinction between motor and vocal tics.
